# Effects of Prehospital Factors on Survival of Out-Of-Hospital Cardiac Arrest Patients: Age-Dependent Patterns

**DOI:** 10.3390/ijerph17155481

**Published:** 2020-07-29

**Authors:** Bo Yoon Rhee, Boram Kim, Yo Han Lee

**Affiliations:** 1 Korea Centers for Disease Control and Prevention, Cheongju 28160, Korea; precious4773@korea.kr (B.Y.R.); borami414@korea.kr (B.K.); 2 Graduate School of Public Health, Ajou University, Suwon 16499, Korea

**Keywords:** out-of-hospital cardiac arrest, survival rates, prehospital factors

## Abstract

Many prehospital factors that are known to influence survival rates after out-of-hospital cardiac arrest (OHCA) have been rarely studied as to how their influence varies depending on the age. In this study, we tried to find out what prehospital factors affect the survival rate after OHCA by age groups and how large the effect size of those factors is in each age group. We used the South Korean OHCA registry, which includes information on various prehospital factors relating OHCA and final survival status. The association between prehospital factors and survival was explored through logistic regression analyses for each age group. The effects of prehospital factors vary depending on the patient’s age. Being witnessed was relatively more influential in younger patients and the presence of first responders became more important as patients became older. While bystander cardiopulmonary resuscitation (CPR) did not appear to significantly affect survival in younger people, use of an automated external defibrillator (AED) showed the largest effect size on the survival in all age groups. Since the pathophysiology and etiologies of OHCA vary according to age, more detailed information on life support by age is needed for the development and application of more specialized protocols for each age.

## 1. Introduction

Out-of-hospital cardiac arrest (OHCA) is a global health problem that affects 55 people per 100,000 people worldwide, but the survival rate varies significantly from community to community [1,2]. Many studies have attempted to identify factors affecting the survival rate after OHCA. Basically, predictors of survival after OHCA can be broadly classified into patient factors, event factors, system factors, and treatment factors [3,4,5,6]. Specifically, a series of actions are needed, including the immediate recognition of the cardiac arrest and the activation of an emergency response system, early cardiopulmonary resuscitation (CPR), rapid defibrillation, effective advanced life support, and integrated treatment after cardiac arrest [7,8]. Past medical literature had focused more on hospitals and advanced life-sustaining treatments than problems related to community care and basic life-sustaining. However, in recent years, awareness of the importance of basic life support (BLS), the role of the community, and the key functions of emergency medical dispatch in coordinating bystander CPR and early defibrillation has increased [9,10].

However, several factors that have been recognized to influence survival rates after OHCA have been rarely studied as to how their influence varies depending on the demographic variables of OHCA patients, especially age. Since the pathophysiology and etiologies of OHCA are different for each age group [11,12,13], it can be expected that factors affecting survival or their influence will differ according to age. The aims of this study were to find out what prehospital factors affect the survival rate after OHCA and how large the effect size of those factors is by age groups based on the data source conducted by the Korea Centers for Disease Control and Prevention (KCDC) for all OHCAs that have occurred in South Korea for two years.

## 2. Materials and Methods

### 2.1. Data Source and Study Population

Since 2006, the South Korean OHCA registry (KOHCAR) of cardiac arrest patients transported through the Emergency Medical Service (EMS) has been built by the KCDC in cooperation with the Central Fire Service (CFS). The EMS run sheet, EMS CPR registration, and dispatch CPR registration were merged into one database by CFS’s EMS quality committee and sent to KCDC. The KCDC cleaned up the hospital information database and reviewed hospital records for inpatient treatment and outcomes [14]. The data used in this study included only those who were confirmed to be OHCA by EMS personnel and were finally identified as OHCA based on the medical records of each patient after being transferred to the hospital. Data obtained from KOHCAR include the date of the OHCA, the patient’s demographic information, the location of the OHCA, the witness of the event, the bystander CPR, application of prehospital automated external defibrillators (AEDs), the etiology of the OHCA, the initial electrocardiographic rhythm, and the experience of return of spontaneous circulation (ROSC), survival to discharge, and cerebral performance categories (CPC) at discharge.

**Figure 1:**
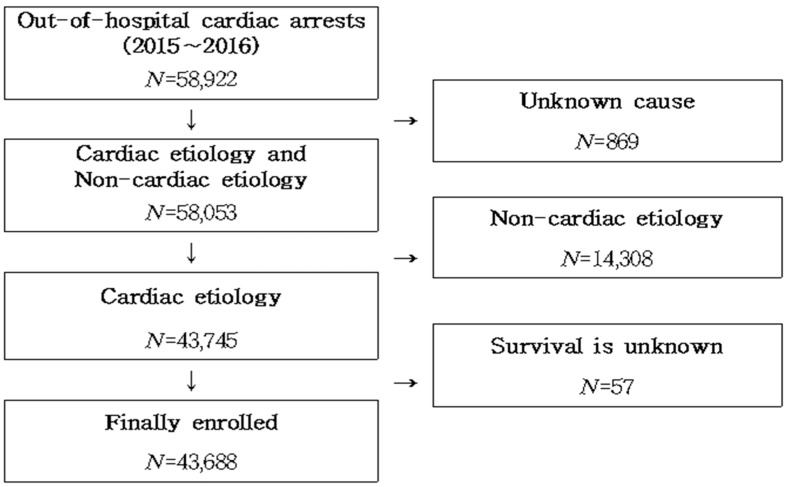
Process for selecting the study population.

The study population of this study initially included all OHCAs that occurred from 1 January 2015 to 31 December 2016. As shown in Figure 1, we first excluded cases where the etiology of OHCA was unclear or not cardiogenic. In addition, we excluded cases in which survival status was unclear, and finally 43,688 cases were selected for the study. The KOHCAR we used was approved by the National Statistical Office as a national statistic (approval number 117088), and is released to the general public through the National Statistical Office (http://kosis.kr). The raw data does not disclose any information that can estimate a specific individual based on the “Personal Information Protection Act” and “Statistics Act”. The institutional review board of the Konyang University Hospital and the KCDC approved the study protocol (KYU-2018-056-01).

### 2.2. Independent and Dependent Variables

**Figure 2:**
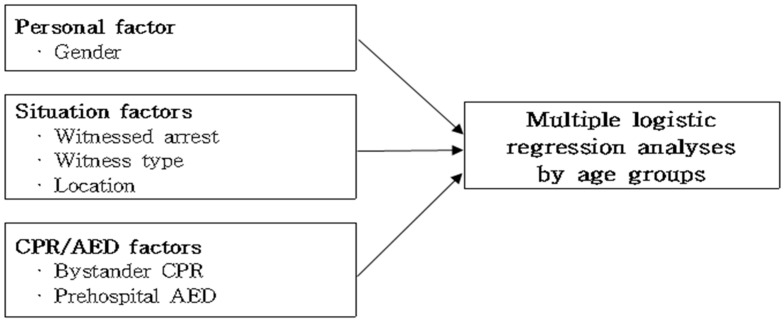
Study framework. CPR, cardiopulmonary resuscitation; AED, automated external defibrillator.

This study basically focused on prehospital factors. The initial electrocardiographic rhythm, ROSC, the response time, and the time of witnessing are important prehospital factors, but many of these items were often missing and recorded as ‘unknown’, so they were not included in the final analysis. We included six prehospital variables with complete data fidelity in the final analytical model. The independent variables were classified into the subject’s personal factors, situation factors, and CPR/AED factors. Gender was used as the personal factor, and whether or not witness, witness type, and place of event were used as the situational factors. CPR/AED factors included whether CPR was performed and whether or not AED was used (Figure 2).

The gender was divided into ‘male’ and ‘female’, and whether or not witnessed were classified as ‘unwitnessed’ and ‘witnessed’. Witness types were divided into ‘first responders’ and the ‘laypeople’. The first responders were those who have essentially completed training on rescue and first aid, including CPR, according to Article 14 (Education on Rescue and First Aid) of the Korean Emergency Medical Care Act, and other witnesses were designated as the layperson. The place where the cardiac arrest occurred was classified as ‘public place’ and ‘non-public place’. Public places include roads/highways, public buildings, leisure-related facilities, industrial facilities, commercial facilities, and transportation terminals. Among the CPR/AED factors, whether CPR was performed by the layperson was classified as ‘yes’ or ‘no’. Likewise, according to whether AED was used before arrival at the hospital, it was also classified as ‘yes’ or ‘no’.

The dependent variable was the survival status. If the patient was transferred to another hospital after the spontaneous circulation was recovered, or if the patient was discharged after the spontaneous circulation was recovered, it was designated as ‘survival’. Those who died or were discharged without hope were classified as ‘death’.

### 2.3. Statistical Analyses

The age groups of the study subjects were divided into six groups as follows: 0–6 years old, 7–18 years old, 19–39 years old, 40–59 years old, 60–79 years old, and 80 years old or older. To understand the general characteristics of the subjects, the frequency and percentage of categorical variables and the mean and standard deviation were used for continuous variables. To compare the survival results according to each factor, a chi-square test was performed on categorical variables and an independent sample *t*-test was performed on continuous variables. We performed multivariate logistic regression analysis for each of the six age groups to understand the relationship with factors affecting survival outcomes (Figure 2). Each analysis was performed with a single model consisting of survival variables and six independent variables. In each Hosmer and Lemeshow goodness-of-fit test of the six analyses, all models were found to be suitable because the *p*-value was greater than 0.05. All tests were treated as statistically significant when the probability of significance was 0.05 or less. SPSS Statistics 20.0 (IBM, Armonk, NY, USA) was used for data analysis.

## 3. Results

Table 1 shows the distribution of variables by study subjects by survival and death after OCHA. Male patients survived 13.7% higher than female patients. By age, the survival proportion for patients aged 7–18 was the highest at 30.7%, and declined with age. Survival proportions were higher in cases where OHCA was witnessed, was seen by a first responder, or occurred in public places, and CPR or AED was performed.

Table 2 shows the results of the multiple logistic regression analysis conducted to identify factors affecting the survival of OHCA by age groups while controlling the effects of other variables. Gender did not appear to be associated with the survival at all ages. However, when witnessed during OHCA, survivable odds were 2.6–8.4 higher than those not witnessed. In particular, in early childhood and adolescence, being witnessed had greater effects on the probability of survival than other age groups. When the witness was the first responder, the relative impact of this on survival increased as the patient’s age increased. Survival odds after OHCA in a public place were higher in all age groups than in a non-public place, especially in early childhood. Whether bystander CPR was performed at the place where OHCA occurred had little effect on survival in younger people, whereas AED application showed significantly high odds ratios at all ages.

## 4. Discussion

### 4.1. Main Findings

In this study, we found that the prehospital factors affecting the survival of OHCA were not just identified, but that the factors related to survival and the size of effects varied according to the age group. Being witnessed was relatively more influential in childhood and younger ages, and being witnessed by first responders became more important as patients became older. The survival rate is high in OHCAs occurring in public places, especially in children and the elderly. On the other hand, bystander CPR did not appear to significantly affect survival in younger people. The factor showing the largest effect size was the use of AED, which showed very large strength of associations in all age groups. In other words, this study showed that the effects of prehospital factors known to be important in surviving of OHCA could vary depending on the patient’s age.

### 4.2. Implications in South Korean Context

In South Korea, nearly 300,000 OHCA occurred every year, and the probability of survival or a good neurological outcome is relatively low compared to other developed countries [15,16,17]. Under the perception of this problem, South Korea has recently taught laypeople to learn how to provide bystander CPR, including the use of AEDs, and has introduced prehospital advanced life support (ALS) by Emergency Medical Services [18]. South Korea has collected OHCA data for the past 14 years, reporting the risk factors of death to the public [19,20]. Risk factors for OHCA deaths include elderly and female patients [21], non-public locations [22], and no witnesses [19,20]. On the other hand, factors that can reduce death include bystander CPR [17,22,23], shockable rhythm [17,23], and prehospital AED use [24,25].

Our findings enrich the knowledge of survival-related factors on OHCA identified in previous studies. Since the pathophysiology and causes of OHCA vary according to age, it is important to know that factors that are expected to increase survival in general may appear ineffective, depending on the patient’s age [26,27,28,29]. Currently, BLS protocols are applied differently to the two groups, children and adults, based on previously identified evidence. More detailed medical and public health information on BLS by age is needed for the development and application of more specialized BLS protocols for each age. For example, the higher the age group, the greater the survival rate if found in public places, by first respondents, or bystander CPR, so the following intervention needs to be considered. In places where people of middle-aged and older gather more, more primary respondents need to be deployed, and more CPR training programs for families supporting the elderly should be implemented. In order to reduce the probability of OHCA occurring when the elderly are alone in a private space such as a home, it may be necessary to further increase the public facilities for the elderly.

The prehospital factors we studied later affect various hospital factors and are also related to the recently recommended extracorporeal cardiopulmonary resuscitation (ECPR). South Korea added new information on ECPR in the field of ALS in the 2015 revised Korean CPR guidelines. When an extracorporeal circulation device is connected, oxygenated blood circulates without chest compressions and artificial respiration. Many indications for ECPR are related to prehospital factors, such as those under 80 years of age, or a refractory OHCA, or bystander CPR [30]. The results of our study show the impact on the survival of prehospital factors by age and can be considered when applying ECPR with age.

In South Korea, there are a total of 429 emergency medical institutions with three hierarchical orders depending on the size of the region. However, the distribution of these institutions is regionally skewed, and there are only a few emergency medical institutions located in rural areas, so regional disparities exist. In particular, the regional variation in establishing a medical infrastructure for specialized emergency diseases such as cardiovascular, mental, and pediatric diseases is more serious. This biased deployment of emergency medical resources increases the regional variation in mortality rates for critically ill patients. As a result, South Korea still has lower numbers of emergency arrivals in a reasonable time and reliability of emergency medical service other developed countries. It is highly likely that emergency patients will survive after receiving the final emergency treatment within the golden hour. In 2018, the rate of trauma patients arriving within 3 h after the onset was only 35.01%. The incidence of traffic accidents is still high, and as the rate of suicide among young people and the elderly ranks first among Organization for Economic Cooperation and Development (OECD) countries, the demand for emergency medical treatment by trauma continues to increase [31].

### 4.3. Reasons for Different Effects for Age

Child subjects in this study were limited to cardiogenic OHCA. Children usually have an unshockable rhythm, asystole, or bradycardia, unlike adults when cardiac arrest occurs [32,33,34]. Therefore, in the case of OHCA in children, ALS is relatively more important than BLS. This seems to be the reason why the effects of the first responder, bystander CPR, and AED application on survival were unclear in our study. While the effect of the bystander CPR or first responder is not clear in school age patients (aged 7–18), the use of AED directly or indirectly through witnesses when an event occurs has been shown to dramatically improve survival. This is probably because many major etiologies of OHCA in the school age are congenital and/or genetic like in children [35,36]. Young adults generally have more OHCA due to coronary artery disease (CAD) than younger groups, and more OHCA due to genetic factors than older groups. Young adults generally have more OHCA due to CAD than younger groups, and more OHCA due to genetic factors than older groups [37,38,39]. Therefore, the patterns of impact of the first responder, bystander CPR, and AED use are located between the younger and the older. OHCA in the elderly, where CAD is the most common cause, shows in our study that the survival rate is lower than that of other age groups, and that several prehospital factors need to operate simultaneously. Taken together, our research shows that the relative importance of BLS to ALS for survival is different because the cause of OHCA is age-dependent, and the survival rate in OHCA due to CAD can only rise if all elements of BLS are performed together.

### 4.4. Limitations

The most serious limitation of this study is that this study reflects a lot of Korean context, so it takes a lot of attention to generalize. Korea has a very limited distribution of primary respondents compared to other developed countries, and their performance is low. In addition, the probability and quality of bystander CPR is relatively low. Therefore, the size of the effect of these factors, which was confirmed to have a great influence on the survival rate in the previous studies, came to be low across all ages in our analysis. Care must be taken to interpret the small effect size of these factors in our analysis.

Direct comparison of odds ratios, especially applied to different groups, requires careful interpretation. Each of the odds ratios presented in our analysis is for only one variable in one age group. However, if this characteristic is sufficiently considered, the relative importance of the variable can be grasped by comparing the odds ratios. For example, the odds ratio of AED use at 19–39 years was 7.82, which was greater than the odds ratio of 5.41 at 40–59 years, indicating that the relative impact of the AED use at the age of 19–39 is greater than that of 40–59 years.

The study did not take into account hospital factors and other Utstein factors [40]. The KOHCAR we used was initially designed to reflect the Utstein Style’s recommendations, but did not include all Utstein factors due to the unfavorable survey conditions in South Korea. Additionally, as mentioned in the methods section, some of the items included in the survey were often missing or marked as unknown, so the failure to use these variables in this study is another major limitation. Therefore, our logistic analysis model cannot be considered as an extensive evaluation of factors affecting the survival rate after OHCA. Most studies of OHCA survival so far, including ours, have focused on finding out what factors influence survival. However, few studies have recently been conducted to quantify the relative impacts of these factors to develop a predictive model of OHCA survival [41,42]. This predictive model can be used for stratifying each patient according to risk. The results of this study, suggesting that factors affecting OHCA survival may differ with age, also need to be included in the survival prediction model.

## 5. Conclusions

This study tried to find out what prehospital factors affect the survival rate after OHCA and how large the effect size of those factors is by age groups using a national OHCA registry. This study showed that the effects of prehospital factors known to be important in surviving of OHCA through the previous studies could vary depending on the patient’s age. Being witnessed was relatively more influential in younger patients and the presence of first responders became more important as patients became older. While bystander CPR did not appear to significantly affect survival in younger people, use of AED showed the largest effect size on the survival in all age groups. Since the pathophysiology and etiologies of OHCA vary according to age, more detailed information on BLS by age is needed for the development and application of more specialized BLS protocols for each age.

## Figures and Tables

**Table 1:** General characteristics of the study population by survival status.

Variables	Total	Survival		Death		*p*-Value ^1^
		*N*	%	*N*	%	
Gender						<0.001
Male	27,268	3733	13.7	23,535	86.3	
Female	16,420	1436	8.7	14,984	91.3	
Age						<0.001
0–6	451	71	15.7	380	84.3	
7–18	231	71	30.7	160	69.3	
19–39	1546	411	26.6	1135	73.4	
40–59	9406	2034	21.6	7372	78.4	
60–79	18,911	2054	10.9	16,857	89.1	
≥80	13,143	528	4.0	12,615	96.0	
Witnessed arrest						<0.001
Witnessed	20,896	3936	18.8	16,960	81.2	
Unwitnessed	17,982	918	5.1	17,064	94.9	
Unknown	4810	315	6.5	4495	93.5	
Witness						<0.001
First responder	3751	861	23.0	2890	77.0	
Layperson	30,383	3177	10.5	27,206	89.5	
Unknown	9554	1131	11.8	8423	88.2	
Location						<0.001
Public place	5444	1313	24.1	4131	75.9	
Non-public place	32,294	2935	9.1	29,359	90.9	
Unknown	5477	876	16.0	4601	84.0	
Bystander CPR						<0.001
No	3644	506	13.9	3138	86.1	
Yes	6827	1366	20.0	5461	80.0	
Not applicable	1855	456	24.6	1399	75.4	
Unknown	31,362	2841	9.1	28,521	90.9	
Prehospital AED use						<0.001
No	2253	151	6.7	2102	93.3	
Yes	5684	2279	40.1	3405	59.9	
Unknown	35,751	2739	7.7	33,012	92.3	
Total	43,688	5169	11.8	38,519	88.2	

^1^ Chi-square test; CPR, cardiopulmonary resuscitation; AEDs, automated external defibrillators.

**Table 2:** Prehospital factors related to survival of out-of-hospital cardiac arrest (OHCA) patients by age groups. Odds ratios (95% confidence interval).

Variables	Age Groups					
	0–6	7–18	19–39	40–59	60–79	80+
Male (vs. Female)	0.79 (0.44–1.42)	1.03 (0.43–2.48)	0.85 (0.63–1.15)	0.89 (0.77–1.02)	0.99 (0.89–1.10)	1.19 (0.99–1.42)
Witnessed (vs. unwitnessed)	7.16 (3.81–13.47)	8.39 (2.59–27.18)	3.45 (2.48–4.81)	3.72 (3.24–4.27)	2.80 (2.47–3.20)	2.62 (2.09–3.30)
Witnessed by first responder (vs. layperson)	0.83 (0.11–6.33)	1.25 (0.21–7.43)	1.41 (0.68–2.92)	1.89 (1.40–2.57)	2.32 (2.01–2.67)	3.48 (2.05–5.91)
Public place (vs. non-public place)	7.43 (2.18–25.35)	1.61 (0.56–4.64)	1.74 (1.22–2.47)	1.55 (1.35–1.79)	2.34 (2.05–2.67)	2.12 (1.56–2.90)
Yes for bystander CPR (vs. no)	0.85 (0.13–5.37)	6.99 (0.40–120.8)	1.34 (0.70–2.57)	1.73 (1.31–2.28)	1.54 (1.21–1.96)	1.68 (1.01–2.81)
Yes for prehospital AED use (vs. no)	7.82 (0.48–127.42)	13.94 (1.96–99.26)	7.82 (3.80–16.07)	5.41 (3.94–7.41)	7.25 (5.45–9.64)	4.26 (2.61–6.94)

CPR, cardiopulmonary resuscitation; AEDs, automated external defibrillators.
